# Supplementary material for: Effectiveness of dexmedetomidine as a premedication for pediatric patients undergoing outpatient dental surgery under general anesthesia-systematic review and meta-analysis
Source: PeerJ. 2025 Mar 31;13:e19216. doi: 10.7717/peerj.19216 (PMC11967424; doi:10.7717/peerj.19216)
Supplement: Supplemental Information 2 [file peerj-13-19216-s002.docx]

**The rationale for conducting the systematic review / meta-analysis;**

Children experienced oral treatment in the clinic were usually accompanied by pain or fear, always showing crying, struggling and anxiety. The fear and anxiety of pediatric patients are usually correlated with increased level of behavior management problems, they can hardly control their emotions and behaviors, and the poor treatment compliance became barriers to the subsequent medical treatment. Moreover, the poor influence of such terrible experience in childhood would continue into adulthood. The rational use of preoperative sedative medications can be a particular effective measure in guaranteeing better compliance and preventing adverse effects. Therefore, comfort measures taken before tooth extraction should draw more attention from anesthesiologist and dental doctors, which should be regarded as normal before dental treatment.

Dexmedetomidine and midazolam are two sedative drugs commonly applied in clinical practice. Midazolam belongs to classical benzodiazepine, a type of psychoactive drug that have relaxing, calming, hypnotic and anterograde amnesia effects by activating the receptor of inhibitory transmitter GABA of the ascending reticular activating system. But the application of midazolam may be associated with bronchial hyperresponsiveness. Dexmedetomidine is an agonist of α2-adrenergic receptors expressed in the central nervous system same as clonidine, which is known for its safety and arousal sedation effects. Compared with other commonly used sedatives like midazolam, propofol, and fentanyl, Dexmedetomidine avoid the risk of respiratory depression and is increasingly widely used in pediatrics.

**The contribution that it makes to knowledge in light of previously published related reports, including other meta-analyses and systematic reviews.**

For children, especially young children, the use of preoperative sedative drugs is usually necessary in order to increase the degree of cooperation and avoid a series of postoperative adverse consequences caused by preoperative anxiety and fear. But for pediatric outpatient oral surgery, the selection of preoperative sedatives is still unclear at present, Dexmedetomidine and midazolam are commonly used sedative drugs, however, it is still controversial whether dexmedetomidine can supply better preoperative sedation and analgesia for children in the oral clinic. There is a lack of systematic summary in relevant fields. Therefore, we conducted this review in order to provide evidence to support clinical decision-making by physicians for premedication of pediatric outpatient general anesthesia surgery.

This review consists of 7 studies and 496 pediatric patients, the sedative effect of dexmedetomidine premedication versus placebo or midazolam in pediatric patients with surgery in oral clinic were compared. The included studies were all RCT studies, and the time span of included studies was approximately from 2006 to 2022, the baseline data including age, gender, weight, anesthesia and operation time of pediatric patients were relatively balanced between the two groups.

Based on the current evidence, we can conclude that the intranasal dexmedetomidine for premedication in child undergoing oral treatment in clinic has a good sedative effect. Children suffered less anxiety and fear when they are separated from their parents, being more cooperative during anesthesia induction, and less likely to have postoperative delirium. In addition, the sedative effect of dexmedetomidine is comparable to that of midazolam, which is less irritating and has a lower incidence of postoperative delirium. We believe that dexmedetomidine is a better choice when sedation is needed. However, RCT studies with larger sample size and longer follow-up period are needed to further evaluate the efficacy and safety of dexmedetomidine for tooth extraction sedation in pediatric patients. RCTs on sedatives for premedication of tooth extraction in pediatric patients are comparatively limited in number. Most of these are RCT studies with small sample size and without follow-up, which varies in quality, and the postoperative adverse reactions are not evaluated as the primary outcome. Therefore, RCT studies with larger sample size and longer follow-up period assessing postoperative adverse reactions are needed to instruct the use of preoperative sedative drugs.
